# Course and Relation of the Facial Vessels—An Anatomical Study

**DOI:** 10.3390/medicina60050805

**Published:** 2024-05-13

**Authors:** Martin Siwetz, Hannes Widni-Pajank, Niels Hammer, Simon Bruneder, Andreas Wree, Veronica Antipova

**Affiliations:** 1 Division of Macroscopic and Clinical Anatomy, Gottfried Schatz Research Center, Medical University of Graz, Auenbruggerplatz 25, A-8036 Graz, Austria; martin.siwetz@medunigraz.at (M.S.); hannes.pajank@stud.medunigraz.at (H.W.-P.); 2 Department of Oral and Maxillofacial Surgery, Klagenfurt Am Wörthersee Clinic, Feschnigstraße 11, A-9020 Klagenfurt am Wörthersee, Austria; 3 Department of Orthopedic and Trauma Surgery, University of Leipzig, D-04103 Leipzig, Germany; 4 Division of Biomechatronics, Fraunhofer Institute for Machine Tools and Forming Technology Dresden, D-09126 Dresden, Germany; 5 Department of Oral and Maxillofacial Surgery, Medical University of Graz, Auenbruggerplatz 5, A-8036 Graz, Austria; simon.bruneder@medunigraz.at; 6 Institute of Anatomy, Rostock University Medical Center, Gertrudenstr. 9, D-18057 Rostock, Germany; andreas.wree@med.uni-rostock.de

**Keywords:** anatomical variation, clinical significance, face anatomy, facial artery, facial vein, labial and angular vessel

## Abstract

*Background and Objectives*: Facial vascular anatomy plays a pivotal role in both physiological context and in surgical intervention. While data exist on the individual course of the facial artery and vein, to date, the spatial relationship of the vasculature has been ill studied. The aim of this study was to assess the course of facial arteries, veins and branches one relative to another. *Materials and Methods*: In a total of 90 halved viscerocrania, the facial vessels were injected with colored latex. Dissection was carried out, the relation of the facial vessels was studied, and the distance at the lower margin of the mandible was measured. Furthermore, branches including the labial and angular vessels were assessed. *Results*: At the base of the mandible, the facial artery was located anterior to the facial vein in all cases at a mean distance of 6.2 mm (range 0–15 mm), with three cases of both vessels adjacent. An angular vein was present in all cases, while an angular artery was only present in 34.4% of cases. *Conclusions*: The main trunk of the facial artery and vein yields a rather independent course, with the facial artery always located anterior to the vein, while their branches, especially the labial vessels, demonstrate a closer relationship.

## 1. Introduction

Accurate knowledge of facial vascular anatomy plays an important role for maxillofacial, plastic-aesthetic, and ENT surgeons whenever performing procedures in the facial region. For surgical reconstruction of facial defects following traumatic injury or tumor resection, a facial artery flap may be used, or the facial vessels may be utilized as a pedicle for microvascular anastomosis [1,2,3,4,5,6]. The portfolio of reconstruction helps treat facial and perinasal defects [5] and defects of the tongue [4] the oral lining, and the oropharynx [2]. In rare cases, even partial [7] or near total [8,9] facial transplantations may be performed in which the facial artery and facial vein are involved as part of vascularized pedicles [10,11,12]. 

Aside from reconstructive surgical procedures, facial vascular anatomy also plays a pivotal role in aesthetic medicine to ensure that injections of fillers or botulinum toxin in aesthetic medicine are performed safely, as intravascular injections may result in severe complications, including tissue necrosis or even blindness [1,13,14,15,16,17]. To minimize such risks and iatrogenic complications, profound anatomical knowledge of vascular regional anatomy is necessary. 

Among the features typical for the facial artery as a main feeder to the viscerocranium [18,19,20,21,22] is its branching of the external carotid artery [23] and its crossing of the stylohyoid and digastric muscle medially [24,25,26,27]. It further traverses the mandible anterior to the masseter muscle directed to the medial angle of the eye, while giving off its branches [28,29]. However, there is plenty of literature describing the different variations of the facial artery regarding its course, its pattern, and its branching [1,3,12,22,26,29,30,31,32,33,34,35,36,37,38,39,40]. 

Less is known about the anatomy of the facial vein, the main drainage for the blood of the face region [41,42]. The facial vein in most studies on the vascular anatomy of the face so far has been ill studied [29,31,33,43,44]. Its course has been described as rather straight from the angular vein in the medial angle of the eye in a latero-caudal direction towards the margin of the mandible [12,42,45,46,47]. The location of the facial vein at the level of the mandible has been described as being dorsal to the anteriormost fibers of the masseter with an overlap of up to 10 mm [42,48]. The branching pattern of the facial vein is described as rather variable [42]. 

Though much is known about the course, pattern, and branching of the facial artery [1,3,12,22,26,29,30,31,32,33,34,35,36,37,38,39,40], the literature is sparse on the facial vein [12,42,48]. Furthermore, there is limited knowledge of the relation between the facial artery and facial vein and their branches in the facial region. A thorough integration of knowledge on the facial vasculature may help further improve the clinical practice regarding surgical reconstructive procedures [8,9,10,11,48], as well as injections of fillers and botulinum toxin [14,15,16,49,50], to further improve patients’ outcomes and reduce iatrogenic complications. 

The aim of this study was to provide information on the relationship between the facial artery and facial vein, as well as their branches, including the labial vessels and angular vessels. 

## 2. Materials and Methods

**Figure 1:**
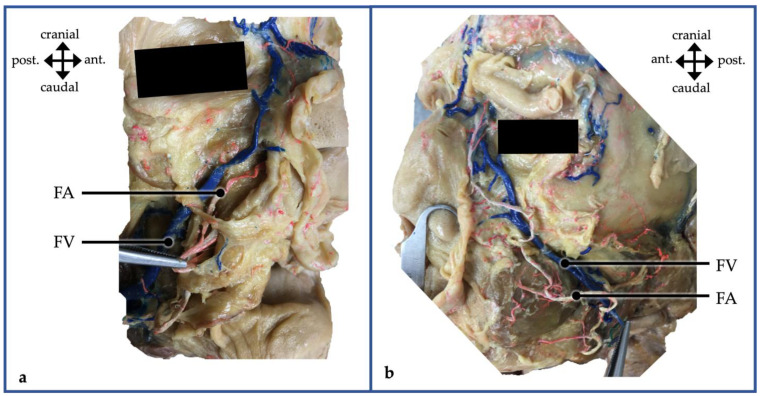
(**a**,**b**) A dissection of the facial vessels is demonstrated for a right (**a**) and left (**b**) hemiviscerocranium of an anatomical specimen. The facial vein (FV) is injected with blue latex and located dorsal to the facial artery (FA), which is injected red. ant., anterior; post., posterior.

For this study, 90 halved viscerocranium specimens were included. Specimens consisted of 43 male specimens (thereof 21 left and 22 right sides) and 47 female specimens (thereof 20 left and 27 right sides). Age at the time of death of the underlying individuals ranged between 39 and 96 years. While alive, all body donors gave their informed consent for the use of their postmortem tissues for research purposes. All body donors were bequeathed to the Division of Macroscopic and Clinical Anatomy of the Medical University of Graz (Austria) under the approval of the ongoing body donation program of the Medical University of Graz and in accordance with the Styrian Death and Funeral Act. All specimens were embalmed using a modified Thiel technique [51,52]. For enhanced visualization and discrimination purposes, the main trunk of the facial artery and facial vein were injected with colored latex in red and blue color, respectively. For this purpose, the vessels were exposed below the level of the mandible, an incision was made in the vessel, and the latex mass was injected via a cannula. The injection mass consisted of 70% distilled water and 30% nature latex GIVUL MR (Fa. Helmut Bergk, Frankfurt/Main, Germany) and was mixed with red color for arterial injection and blue color for venous injection (Figure 1). 

Specimens were only included if, upon visual inspection and further dissection, they were void of major pathological lesions, including tumors or surgical intervention. Furthermore, vessels were only included in the data acquisition if the condition of the tissues and the completeness of latex filling allowed for data acquisition without potential errors. 

Further dissection was performed similar to the approach published in a recent study by our group [42] in a way that after the main trunk of the vessels was dissected and injected with latex, the skin was incised laterally, anterior to the auricle. Skin flaps were elevated medially. Thereafter, the injected facial artery and vein were dissected to the level of the mandible, and the main vessels and their branches were carefully dissected cranially, ensuring not to damage any smaller branches. 

**Figure 2:**
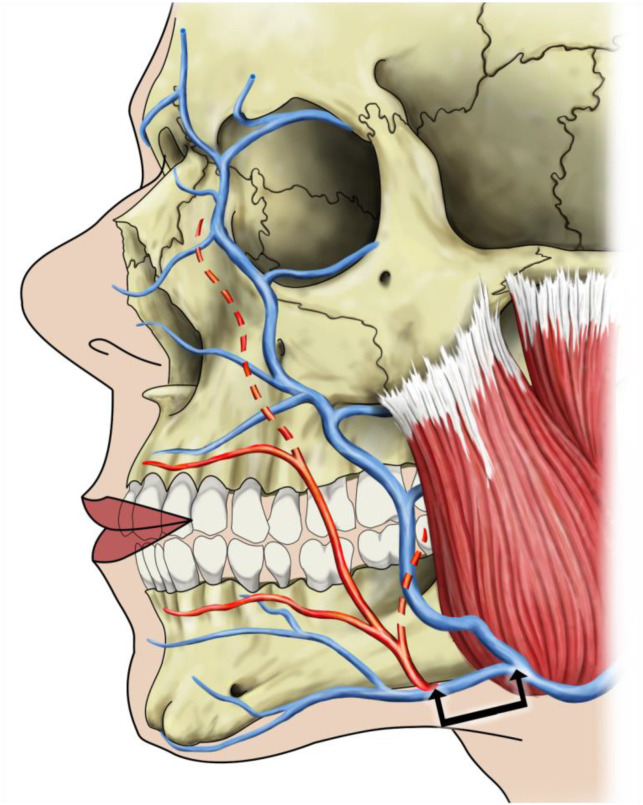
A schematic depiction of the course of the facial artery (red color) and facial vein (blue color) in a left hemiface. The black arrows mark the measurement taken between the facial artery and vein at the base of the mandible.

Here, the relationship between the facial artery and vein was described. For the main stems of the facial artery and facial vein, their distance at the inferior margin of the mandible was measured using calipers, and the results were rounded to whole millimeters. Descriptive statistics were deployed for the distance between the facial artery and vein; for the other vessels, relations were described qualitatively (Figure 2). 

## 3. Results

### 3.1. The Facial Artery Is Always Located Anterior to the Facial Vein 

Data on the spatial relations between the facial artery and facial vein at the inferior margin of the mandible were assessed in 90 hemiviscerocrania. Overall, the facial artery was consistently located anterior to the facial vein. Only in three cases (3%) were the artery and vein in direct contact, so the distance between the two was zero. Overall, the distance measured between the facial artery and vein averaged 6.2 mm (0–15 mm). 

The entire group was then divided into two subgroups based on gender. The female subgroup consisted of 20 left and 27 right half-faces. The average distance between the facial artery and vein was 5.8 mm (0–13 mm), with 7.3 mm (0–13 mm) for left sides and 4.8 mm (2–8 mm) for right sides. 

In the male subgroup, the average distance between the facial artery and vein was 6.5 mm (0–15 mm), with 6.8 mm (0–10 mm) for the left and 6.2 mm (2–15 mm) for the right side. An overview of the distances can be found in Table 1. 

### 3.2. Facial Artery Demonstrated a More Variable Course Than the Vein 

In all cases, the facial artery was located anterior to the vein and consistently demonstrated a more variable course than the vein. With relations at the inferior margin of the mandible as described above, the two vessels diverged in the buccal region (Figure 1). Here, while the facial vein took a rather straight direction, the artery took a more tortuous course. With the main vessels taking a rather independent course, smaller branches were seen to take a course in which the arterial and venous branches come fairly close to each other. 

### 3.3. Labial Arteries Branched off Medial to the Labial Veins 

The relation of the labial vessels was assessed in 74 half-faces. 

In 74 cases, the superior and inferior labial artery branched off the facial artery medial to the vein and took a rather horizontal course. The superior labial vein in 58 cases (78.4%) took a laterocranial direction, reaching the facial vein superior to the branching of the superior labial artery (Figure 2). In 16 cases (21.6%), the superior labial vein took a laterocaudal direction and, following the crossing of the superior labial artery, reached the facial vein below the branching of the superior labial artery. 

**Figure 3:**
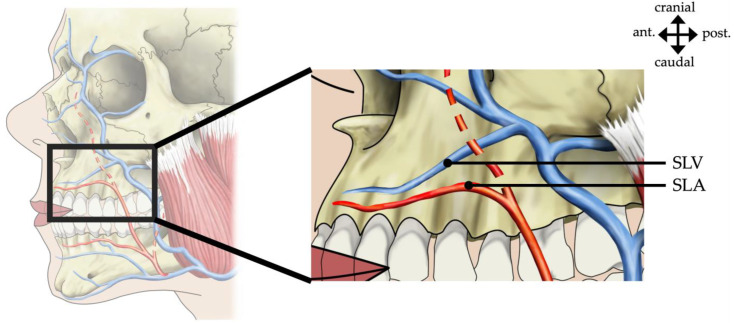
A schematic depiction is shown for the relation of the labial vessels. The superior labial artery (SLA) branches off the facial artery medial to the vein and takes a horizontal course, while the superior labial vein (SLV) takes a laterocranial course. ant., anterior; post., posterior.

While the facial artery and vein in their course were separated, the superior and inferior labial vessels converged as they approached the midline (Figure 3). 

### 3.4. Angular Veins Were Present More Often Than Angluar Arteries 

**Figure 4:**
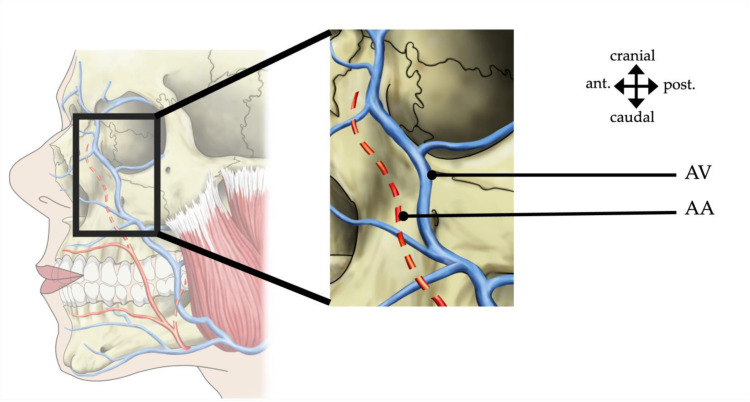
A schematic representation of the relation of the angular artery (AA) and vein (AV), with the dashed line indicating the relatively sporadic presence of the angular artery. ant., anterior; post., posterior.

In all 90 hemiviscerocrania, an angular vein was present, while an angular artery was only found in 31 (34.4%) hemiviscerocrania. The facial artery and vein took a separate course in the buccal region. At the level of the nose, they started to take a course in angular direction, approaching each other and showing a very narrow course (Figure 4). 

## 4. Discussion

### 4.1. Facial Artery Types Are Well Described as Opposed to the Facial Vein 

The contemporary literature entails a large amount of data resulting from dissection-based and angiography-based studies on the branching pattern and type classification of the facial artery. In most cases, such categorization is performed by the termination of the facial artery and its branching pattern [1,3,12,21,26,29,30,31,32,35,36,37,40,53]. Typical categories include a labial type, nasal type, and angular type [3,12,21,30,35,39]. However, an attempt has been made to categorize the facial vessels based on their depth and relation to the facial and mimic musculature [53]. In a previous study, a roundup of the different types of facial artery branching could be shown [22]. 

The course of the vein has been described as being straight from the medial angle of the eye to the lower margin of the mandible with very little variation [12,47,48,54]. However, in a previous study assessing the tributaries of the facial vein, a rather large variability and the possibility of classification based on these vessels could be shown [42]. 

### 4.2. Facial Vein Crosses the Mandible 20 mm Anterior to the Mandibular Angle

Koh and colleagues assessed the facial artery in 47 anatomical specimens and obtained the distance between the facial artery and the stomion at the level of the oral commissure, averaging 44.8 mm. Furthermore, measurements were taken for the facial artery at the base of the mandible, averaging 27 mm anterior to the angle of the mandible [31]. This can be backed by Koziej and colleagues, who, in their computed tomography-based study, obtained the location of the facial artery from various reference points in a detailed fashion and found that the facial artery was located 27.2 mm (median) anterior to the mandibular angle [1]. When comparing their findings to the given results, the facial artery is located on average 6.2 mm anterior to the facial vein. It can, therefore, be deduced that the facial vein is located approximately 20 mm anterior to the mandibular angle. 

As little is known about the facial vein in general, this can also be said about the location of the facial vein in the face. Cotofana and colleagues found the facial vein to be located 0.2–1.0 cm dorsal to the anteriormost fibers of the masseter at the base of the mandible [48]. These findings were backed by a previous study, in which the facial vein was located on average 3.2 mm dorsal to the anterior margin of the masseter [42]. 

### 4.3. Facial Vein Located in a Predictable Position

In their dissection-based study, Lohn and colleagues observed the relation of the facial artery and the facial vein based on 112 specimens. They classified the facial artery based on six different types, with the lateral-nasal type being the most frequent. Regarding the relationship between the facial artery and vein, the vein was proven to be predictable in position posterior to the artery at the base of the mandible and took a direct course to the inner canthus [12]. This can be backed by our findings that show that the facial vein was always located posterior to the artery at a distance of 6.2 mm on average. This predictability is of special importance for the reconstruction of traumatic injuries or when tumor resection facial artery flaps are used. In these cases, the facial artery and vein are used as vascularized pedicles of these flaps; therefore, predictability in location helps with identification and facilitation [10,11,12]. 

Furthermore, the predictability in the location and course of the facial vessels helps to establish safe zones for the injection of fillers or Botox. Injection in these safe zones should help reduce complications caused by accidental intravascular injections [1,13,14,15,16]. 

In addition, based on the findings of this study, it can be said that the main trunks of the facial vessels show a rather independent course, while the smaller branches show a close relation one to another. 

## 5. Limitations

This given study was performed on embalmed tissues with chemical fixatives used, leading to tissue acellularization and degreasing [52]; furthermore, there may be a change in tissue pressure when comparing embalmed to non-embalmed and vital human tissues. This may affect the here measured distances. Furthermore, the number of specimens used for this study is limited; this is especially important when considering that body proportions do influence the measured distances, leading to the possibility of an inadequate depiction of the extremes in a limited number of specimens. Therefore, further studies with large sample sizes, including fresh-frozen tissues, are needed to further back up our findings. 

## 6. Conclusions and Clinical Significance 

This study provides information on the relations of the facial artery and facial vein, as well as their branches, including the labial vessels and angular vessels. It shows that the facial artery is always located anterior to the facial vein, and both vessels diverge in the buccal region. The angular vein was always present, while the angular artery was only present in 34.4% of cases. The position and course of the facial vessels are rather predictable, which may help with the planning of reconstructive surgeries of the face and aesthetic procedures. Furthermore, these results may help radiologists interpret facial vascular anatomy in angiographies. Therefore, these results may help improve patients’ outcomes due to a better understanding of the anatomy. 

## Figures and Tables

**Table 1:** A detailed overview of the distance between the facial artery and vein depicted for sex and side.

Sex	Side	Average	Range
Female	left	7.3 mm	0–13 mm
	right	4.8 mm	2–8 mm
Male	left	6.8 mm	0–10 mm
	right	6.2 mm	2–15 mm

## Data Availability

Data are contained within the article.

## References

[1] Koziej M., Trybus M., Hołda M., Polak J., Wnuk J., Brzegowy P., Popiela T., Walocha J., Chrapusta A. (2019). Anatomical Map of the Facial Artery for Facial Reconstruction and Aesthetic Procedures. Aesthet. Surg. J..

[2] Khan K., Hinckley V., Cassell O., Silva P., Winter S., Potter M. (2013). A Novel Use of the Facial Artery Based Buccinator Musculo-Mucosal Island Flap for Reconstruction of the Oropharynx. J. Plast. Reconstr. Aesthet. Surg..

[3] Dupoirieux L., Plane L., Gard C., Penneau M. (1999). Anatomical Basis and Results of the Facial Artery Musculomucosal Flap for Oral Reconstruction. Br. J. Oral. Maxillofac. Surg..

[4] Joseph S.T., Naveen B.S., Mohan T.M. (2017). Islanded Facial Artery Musculomucosal Flap for Tongue Reconstruction. Int. J. Oral. Maxillofac. Surg..

[5] Camuzard O., Foissac R., Georgiou C., Andot L., Alcaraz F., Baqué P., Bronsard N., Poissonnet G. (2015). Facial Artery Perforator Flap for Reconstruction of Perinasal Defects: An Anatomical Study and Clinical Application. J. Craniomaxillofac. Surg..

[6] Rabbani C.C., Lee A.H., Desai S.C. (2022). Facial Artery Musculomucosal Flap Operative Techniques. Plast. Reconst. Surg..

[7] Duisit J., Maistriaux L., Gerdom A., Vergauwen M., Gianello P., Behets C., Lengelé B. (2018). Nose and Lip Graft Variants: A Subunit Anatomical Study. Plast. Reconst. Surg..

[8] Siemionow M., Papay F., Alam D., Bernard S., Djohan R., Gordon C., Hendrickson M., Lohman R., Eghtesad B., Coffman K. (2009). Near-Total Human Face Transplantation for a Severely Disfigured Patient in the USA. Lancet.

[9] Alam D.S., Papay F., Djohan R., Bernard S., Lohman R., Gordon C.R., Hendrickson M., Siemionow M. (2009). The Technical and Anatomical Aspects of the World’s First Near-Total Human Face and Maxilla Transplant. Arch. Facial Plast. Surg..

[10] Hettiaratchy S., Butler P.E. (2002). Face Transplantation—Fantasy or the Future?. Lancet.

[11] Morris P.J., Bradley J.A., Doyal L., Earley M., Hagan P., Milling M., Rumsey N. (2004). Facial Transplantation: A Working Party Report from the Royal College of Surgeons of England. Transplantation.

[12] Lohn J.W.G., Penn J.W., Norton J., Butler P.E.M. (2011). The Course and Variation of the Facial Artery and Vein: Implications for Facial Transplantation and Facial Surgery. Ann. Plast. Surg..

[13] Lazzeri D., Agostini T., Figus M., Nardi M., Pantaloni M., Lazzeri S. (2012). Blindness Following Cosmetic Injections of the Face. Plast. Reconstr. Surg..

[14] Alam M., Kakar R., Dover J.S., Harikumar V., Kang B.Y., Wan H.T., Poon E., Jones D.H. (2021). Rates of Vascular Occlusion Associated with Using Needles vs Cannulas for Filler Injection. JAMA Dermatol..

[15] Sito G., Manzoni V., Sommariva R. (2019). Vascular Complications after Facial Filler Injection: A Literature Review and Meta-Analysis. J. Clin. Aesthet. Dermatol..

[16] Woodward J., Khan T., Martin J. (2015). Facial Filler Complications. Facial Plast. Surg. Clin. N. Am..

[17] Smit J., Ruhe P., Acosta R., Kooloos J., Hartman E. (2009). The Nasolabial Fold as Potential Vascular Receptor Site: An Anatomic Study. J. Reconstr. Microsurg..

[18] Meegalla N., Sood G., Nessel T.A., Downs B.W. (2021). Anatomy, Head and Neck, Facial Arteries. StatPearls.

[19] Soikkonen K., Wolf J., Hietanen J., Mattila K. (1991). Three Main Arteries of the Face and Their Tortuosity. Br. J. Oral. Maxillofac. Surg..

[20] von Arx T., Tamura K., Yukiya O., Lozanoff S. (2018). The Face—A Vascular Perspective. A Literature Review. Swiss Dent. J..

[21] Bayram S.B., Kalaycioglu A. (2010). Branching Patterns of Facial Artery in Fetuses. New J. Med..

[22] Siwetz M., Turnowsky N., Hammer N., Pretterklieber M., Wree A., Antipova V. (2021). A Rare Case of Facial Artery Branching—A Review of the Literature and a Case Report with Clinical Implications. Medicina.

[23] Pretterklieber M.L., Krammer E.B., Mayr R. (1991). A Bilateral Maxillofacial Trunk in Man: An Extraordinary Anomaly of the Carotid System of Arteries. Cells Tissues Organs.

[24] Stathakios J., Carron M.A. (2021). Anatomy, Head and Neck, Neck Triangle. StatPearls.

[25] Isolan G.R., Rowe R., Al-Mefty O. (2007). Microanatomy and Surgical Approaches to the Infratemporal Fossa: An Anaglyphic Three-Dimensional Stereoscopic Printing Study. Skull Base.

[26] Loukas M., Hullett J., Louis R.G., Kapos T., Knight J., Nagy R., Marycz D. (2006). A Detailed Observation of Variations of the Facial Artery, with Emphasis on the Superior Labial Artery. Surg. Radiol. Anat..

[27] Rao S.B., Vollala V.R., Rao M., Samuel V.P., Deepthinath D., Nayak S., Pamidi N. (2020). Unusual Position of External Carotid Artery: A Case Report. Indian. J. Plast. Surg..

[28] Drenckhahn D., Zenker W. (1994). Benninghoff Anatomie Makroskopische Anatomie, Embryologie Und Histologie Des Menschen.

[29] Pilsl U., Anderhuber F., Neugebauer S. (2016). The Facial Artery-The Main Blood Vessel for the Anterior Face?. Dermatol. Surg..

[30] Furukawa M., Mathes D.W., Anzai Y. (2013). Evaluation of the Facial Artery on Computed Tomographic Angiography Using 64-Slice Multidetector Computed Tomography: Implications for Facial Reconstruction in Plastic Surgery. Plast. Reconstr. Surg..

[31] Koh K.S., Kim H.J., Oh C.S., Chung I.H. (2003). Branching Patterns and Symmetry of the Course of the Facial Artery in Koreans. Int. J. Oral. Maxillofac. Surg..

[32] Lasjaunias P., Berenstein A., Doyon D. (1979). Normal Functional Anatomy of the Facial Artery. Radiology.

[33] Lee H.J., Won S.Y., O J., Hu K.S., Mun S.Y., Yang H.M., Kim H.J. (2018). The Facial Artery: A Comprehensive Anatomical Review. Clin. Anat..

[34] Marx C., Kumar P., Reddy S., Vollala V.R. (2008). Bilateral Variation of Facial Artery: A Case Report. Rom. J. Morphol. Embryol..

[35] Midy D., Mauruc B., Vergnes P., Caliot P. (1986). A Contribution to the Study of the Facial Artery, Its Branches and Anastomoses; Application to the Anatomic Vascular Bases of Facial Flaps. Surg. Radiol. Anat..

[36] Mitz V., Ricbourg B., Lassau J.P. (1973). Facial branches of the facial artery in adults. Typology, variations and respective cutaneous areas. Ann. Chir. Plast..

[37] Niemann K., Lazarus L., Rennie C. (2019). An Anatomical Study of the Facial Artery. Int. J. Morphol..

[38] Padur A.A., Kumar N. (2019). Unusual Branching Pattern and Termination of Facial Artery and Its Clinical Implications for Facial Operations. J. Vasc. Bras..

[39] Pinar Y.A., Bilge O., Govsa F. (2005). Anatomic Study of the Blood Supply of Perioral Region. Clin. Anat..

[40] Vasudha T.K., Divya Shanthi D., Sadashivana G. (2018). A study on course and variations of facial artery on the face. Int. J. Anat. Res..

[41] Bondaz M., Ricard A.-S., Majoufre-Lefebvre C., Caix P., Laurentjoye M. (2014). Facial Vein Variation: Implication for Facial Transplantation. Plast. Reconstr. Surg.-Glob. Open.

[42] Siwetz M., Widni-Pajank H., Hammer N., Pilsl U., Bruneder S., Wree A., Antipova V. (2023). The Course and Variation of the Facial Vein in the Face—Known and Unknown Facts: An Anatomical Study. Medicina.

[43] Carruthers J.D. (2014). Discussion: New Anatomical Insights on the Course and Branching Patterns of the Facial Artery. Plast. Reconst. Surg..

[44] Hong S.J., Park S.E., Jo J.W., Jeong D.S., Choi D.S., Won J.H., Hwang M., Kim C.Y. (2020). Variant Facial Artery Anatomy Revisited: Conventional Angiography Performed in 284 Cases. Medicine.

[45] Tandler J. (1926). Lehrbuch Der Systematischen Anatomie. Das Gefäss-System.

[46] Luschka H. (1865). Die Anatomie Der Glieder Des Menschen.

[47] Houseman N.D., Taylor G.I., O A., Pan W.-R. (2000). The Angiosomes of the Head and Neck: Anatomic Study and Clinical Applications. Plast. Reconstr. Surg..

[48] Cotofana S., Steinke H., Schlattau A., Schlager M., Sykes J.M., Roth M.Z., Gaggl A., Giunta R.E., Gotkin R.H., Schenck T.L. (2017). The Anatomy of the Facial Vein: Implications for Plastic, Reconstructive, and Aesthetic Procedures. Plast. Reconst. Surg..

[49] Calva D., Chopra K.K., Sosin M., De La Cruz C., Bojovic B., Rodriguez E.D., Manson P.N., Christy M.R. (2015). Manson’s Point: A Facial Landmark to Identify the Facial Artery. J. Plast. Reconstr. Aesthet. Surg..

[50] Beleznay K., Carruthers J.D.A., Humphrey S., Jones D. (2015). Avoiding and Treating Blindness from Fillers: A Review of the World Literature. Dermatol. Surg..

[51] Thiel W. (2002). Supplement to the conservation of an entire cadaver according to W. Thiel. Ann. Anat..

[52] Hammer N., Löffler S., Bechmann I., Steinke H., Hädrich C., Feja C. (2015). Comparison of Modified Thiel Embalming and Ethanol-Glycerin Fixation in an Anatomy Environment: Potentials and Limitations of Two Complementary Techniques: Modified Thiel Complements Ethanol Fixation. Anat. Sci. Educ..

[53] Lee J.G., Yang H.M., Choi Y.J., Favero V., Kim Y.S., Hu K.S., Kim H.J. (2015). Facial Arterial Depth and Relationship with the Facial Musculature Layer. Plast. Reconstr. Surg..

[54] Zhou W., Wan L., Zhang P., Yuan H., Jiang H., Du Y. (2017). Anatomical Study and Clinical Application of Facial Artery Perforator Flaps in Intraoral Reconstruction: Focusing on Venous System. J. Oral. Maxillofac. Surg..

